# When Time Equals Vision: The Neuro-Ophthalmic Outcomes of Patients with Fulminant Idiopathic Intracranial Hypertension Undergoing Emergent Cerebral Transverse Venous Stenting

**DOI:** 10.3390/brainsci15101099

**Published:** 2025-10-13

**Authors:** Assaf Kratz, Eyal Walter, Asaf Honig, Alexander Chorny, Gal Ben-Arie, Erez Tsumi, Tamir Regev, Anat Horev

**Affiliations:** 1 Department of Ophthalmology, Soroka University Medical Center, Beer-Sheva 84101, Israel; 2 Department of Neurology, Soroka University Medical Center, Beer-Sheva 84101, Israel; 3 Department of Radiology, Soroka University Medical Center, Beer-Sheva 84101, Israel

**Keywords:** idiopathic intracranial hypertension, fulminant, cerebral transverse venous stenting

## Abstract

**Background:** Fulminant idiopathic intracranial hypertension (IIH) is a rare and vision-threatening variant of IIH, characterized by rapid visual deterioration and a high risk of irreversible blindness. Urgent intervention is required to prevent permanent optic nerve damage. Cerebral transverse venous stenting (CTVS) has emerged as an effective treatment for medically refractory IIH, but data on its use in fulminant cases remain limited. **Methods:** A retrospective consecutive cohort study was conducted at a tertiary center and included all patients with fulminant IIH diagnosed by modified Dandy criteria, with bilateral transverse sinus stenosis > 50% and a trans-stenotic pressure gradient ≥ 8 mmHg on venography. Before stenting, patients received high-dose acetazolamide (up to 3000 mg/day) and IV methylprednisolone (1000 mg/day × 3). Neuro-ophthalmic assessment included BCVA, Ishihara color vision, pupillary exam, disc edema grading, Humphrey visual fields, and optical coherence tomography (OCT). Follow-up occurred at baseline (admission), 1 week, 1 month, 3 months, and 12 months. **Results:** Five young female patients underwent successful CTVS without peri- or post-procedural complications. Significant improvement in headache and stabilization or recovery of visual function were observed in all patients. OCT revealed early retinal nerve fiber layer thinning within one week, preceding clinical resolution of papilledema. **Conclusions:** Emergent CTVS appears to be a safe and effective vision-preserving procedure in fulminant IIH, offering rapid intracranial pressure reduction and early neuro-ophthalmologic improvement. OCT may serve as a useful early predictor of treatment success, supporting its role in post-procedural monitoring. Larger prospective studies are warranted.

## 1. Introduction

Idiopathic Intracranial Hypertension (IIH) predominantly affects overweight women of childbearing age, typically presenting acutely with headaches and visual disturbances resulting from elevated intracranial pressure (ICP) [1,2]. The principal source of long-term morbidity is progressive optic neuropathy, believed to arise from chronic axonal stasis secondary to persistently elevated ICP. While moderate vision loss may be at least partially reversible with resolution of disc edema, chronic papilledema often leads to irreversible optic neuropathy and permanent visual field defects [1,2].

When vision loss in IIH progresses rapidly, posing an imminent risk of blindness, the condition is referred to as “fulminant” IIH. Thambisetty et al. defined fulminant IIH by three criteria: (1) acute onset of symptoms and signs of intracranial hypertension, (2) progression to severe visual loss within four weeks of symptom onset, and (3) rapid visual deterioration over a few days despite prompt administration of appropriate medical therapy [3].

To prevent irreversible blindness, there is broad consensus that urgent surgical intervention is required [4]. The most widely accepted vision-preserving procedures are optic nerve sheath fenestration (ONSF) and cerebrospinal fluid (CSF) diversion through surgical shunting, most commonly a ventriculoperitoneal (VP) shunt. Both interventions are considered effective in halting vision loss but carry substantial short- and long-term complication risks [5,6].

Since Higgins first described venous sinus stenting for IIH in 2002, accumulating evidence has supported the use of cerebral transverse venous stenting (CTVS) as an elective procedure, with consistently favorable outcomes [7,8,9].

This study reports the neuro-ophthalmological outcomes of patients with fulminant IIH who underwent emergent CTVS (ECTVS) at a single tertiary medical center.

## 2. Materials and Methods

This retrospective cohort study was conducted at Soroka University Medical Center (SUMC), Israel. All consecutive patients diagnosed with fulminant IIH who underwent emergent cerebral transverse venous stenting (ECTVS) during the study period were included. All patients presenting with suspected IIH underwent urgent venous imaging, either computed tomography (CT) venography or magnetic resonance (MR) venography, to exclude cerebral venous sinus thrombosis. The diagnosis of IIH was established using the modified Dandy criteria. Following admission, patients received medical therapy and were closely monitored for treatment response.

We adopted a deliberately restrictive definition of fulminant IIH to capture imminently vision-threatening cases suitable for emergent stenting. Specifically, inclusion required rapid visual deterioration within days, florid papilledema (Frisen grade ≥ 3), and failure to stabilize despite high-dose acetazolamide and IV steroids, together with HVF criteria (MD ≤ 15 dB and an additional decline ≥ 5 dB shortly after presentation). This approach prioritizes clinical urgency and cohort homogeneity relative to earlier, broader definitions [3].

Per institutional protocol, all patients with severe disc edema and profound vision loss were treated with high-dose acetazolamide (up to a maximally tolerated daily dose of 3000 mg) and a 3-day course of intravenous (IV) methylprednisolone (1000 mg daily).

Fulminant IIH was defined as rapid visual deterioration occurring within days of symptom onset. Severe and rapidly progressive visual loss was defined as both a Humphrey Visual Field (HVF; Humphrey Field Analyzer 3, Carl Zeiss Meditec, Inc., Dublin, CA, USA) mean deviation (MD) of ≤15 dB and a subsequent decline of ≥5 dB shortly after presentation. In patients unable to undergo HVF testing because of incapacitating headache, severe visual loss was determined by confrontation fields, which were required to show constriction to within 10° of fixation. Vision loss in fulminant IIH was required to occur in the context of florid disc edema (Frisen grade ≥ 3) and to demonstrate progression despite appropriate medical treatment per the above protocol.

All venous imaging studies were jointly reviewed by a neuroradiologist (IS, GBA) and an interventional neurologist (AH). Patients with hemodynamically significant bilateral transverse sinus stenosis (>50% luminal narrowing) were offered ECTVS. During venography, a trans-stenotic pressure gradient ≥ 8 mmHg was considered as the institutional cutoff for stent deployment (Precise 7 × 40 or 8 × 40, Cordis, Hialeah, FL, USA), reflecting prior reports and local expert consensus.

After informed consent was obtained, all patients who elected to undergo CTVS were treated by a single endovascular neurologist (AH) and were included in the cohort.

Each patient underwent a comprehensive neuro-ophthalmological evaluation, including documentation of best-corrected visual acuity (BCVA), color vision (Ishihara plates), pupillary reactivity, ocular motility, grading of disc edema, computerized HVF testing, and optical coherence tomography (OCT; Spectralis, Heidelberg Engineering, Heidelberg, Germany). At each visit, OCT was performed with peripapillary retinal nerve fiber layer (RNFL) measurements. In cases where RNFL thickness could not be measured automatically due to severe disc edema and signal degradation, layer segmentation was manually corrected by an experienced neuro-ophthalmologist (EW). Patients were assessed within 24 h of hospital admission, during hospitalization and prior to ECTVS, and re-evaluated at 1 week, 1 month, 3 months, and 12 months following stent placement.

Institutional Review Board (IRB) approval was obtained for prospective data collection into the institutional IIH database and for the retrospective database review included in this study, with a waiver of informed consent (institutional approval number 0044-20).

The IRB approved a waiver of informed consent for this retrospective chart review. To protect confidentiality, all records were de-identified before analysis and assigned unique study codes; the linkage file was stored separately on a secure institutional server. Access to the dataset was limited to authorized study personnel. Data were stored on password-protected, encrypted institutional systems, and only aggregate results are reported.

## 3. Results

During the study period, five consecutive patients were diagnosed with fulminant IIH and underwent emergent CTVS. Clinical data at presentation are summarized in Table 1. All patients were young females aged 17–21 years. One patient met criteria for severe obesity (body mass index [BMI] > 40), two were obese (30 < BMI < 40), one was overweight (25 < BMI < 30), and one had a healthy BMI (19 < BMI < 25); the average BMI was 32.8 ± 10.4. All patients presented with severe headache and visual disturbances.

Opening pressure, measured in the lateral decubitus position, ranged from 380 to 510 mm H_2_O (mean 454.0 ± 67.7 mm H_2_O). All patients were rapidly titrated to the highest tolerated dose of acetazolamide (up to 3000 mg daily). One patient (patient 4) was intolerant of acetazolamide side effects, declined treatment, and was managed with high-dose furosemide (80 mg) as an alternative. All patients also received intravenous steroids per institutional protocol. Medical therapy prior to ECTVS is detailed in Table 1.

Table 2 summarizes visual acuity, color vision, disc edema grading, HVF parameters, and OCT measurements. Presentation data correspond to the last neuro-ophthalmologic examination prior to ECTVS.

No peri- or post-procedural complications occurred. All patients reported marked improvement in headache and subjective visual symptoms within days following the procedure. The mean follow-up duration was 12.8 months (range: 6–26 months).

No patient experienced a decrease in BCVA after ECTVS. Four eyes of three patients had BCVA worse than logMAR 0.3 (Snellen 20/40) at presentation; all showed improvement by the one-week follow-up. By the final visit, only one eye (patient 5, right eye) had BCVA worse than logMAR 0.3. This eye was the only one presenting with a relative afferent pupillary defect (RAPD), which persisted throughout follow-up.

Color vision, tested with Ishihara plates, was preserved in all patients, and no dyschromatopsia was detected despite the presence of significant optic disc edema.

The mean pre-procedure visual field mean deviation (MD) was −23.0 ± 10.7 dB. Two patients (patients 1 and 2) could not undergo HVF testing initially and were assessed clinically by confrontation fields. HVF performed one week after ECTVS showed no significant early change in MD. Over the following weeks, gradual improvement was observed in all patients, reaching a plateau between 1–3 months post-procedure. The mean MD at last follow-up was −12.0 ± 10.0 dB, representing an average improvement of 11.0 ± 8.8 dB (*p* = 0.002) compared with pre-ECTVS testing.

**Figure 1:**
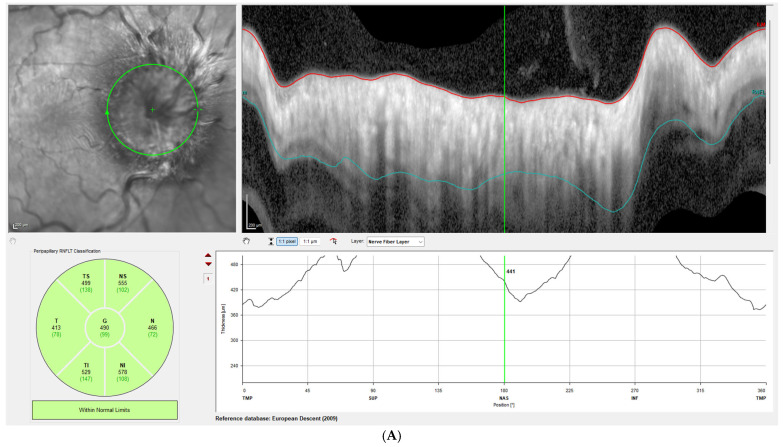

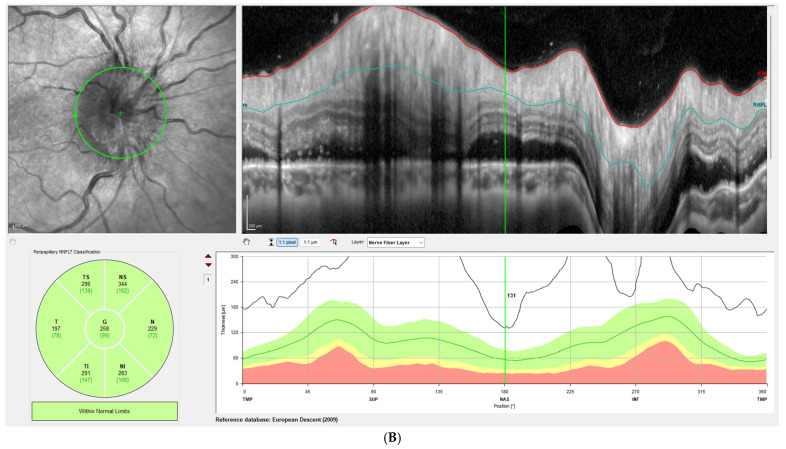
Right-eye OCT RNFL measurements of patient 3 at initial presentation, before ECTVS (**A**) and three days after the procedure (**B**). The green circle indicates the circle along which the RNFL thickness is measured. The red and turquoise lines represent the estimated borders of the RNFL layer.

**Figure 2:**
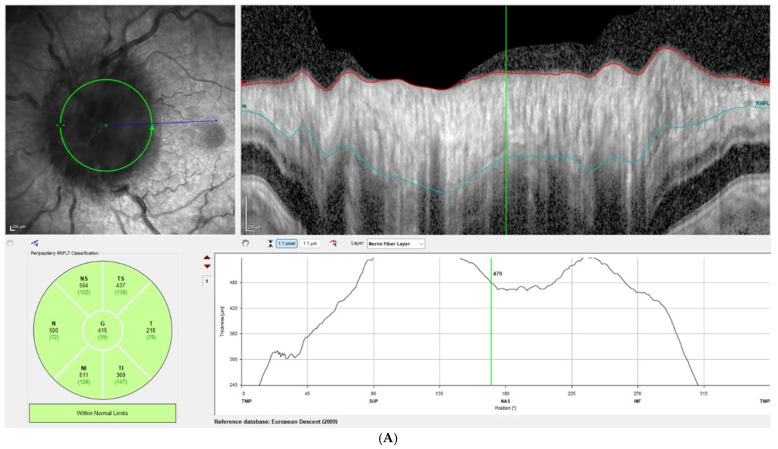

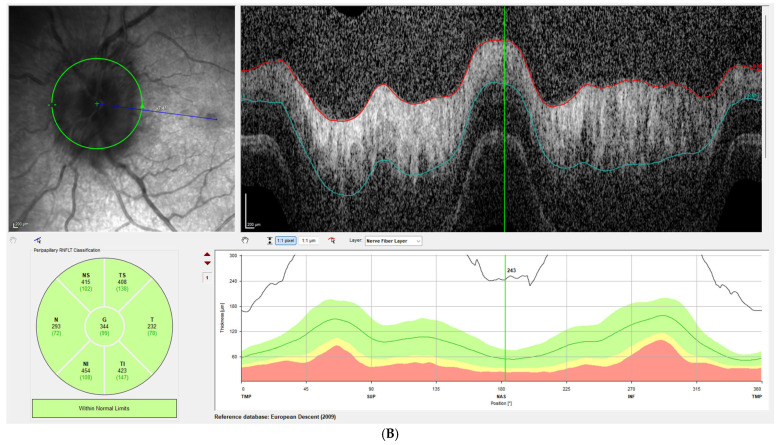
Left-eye OCT RNFL measurements of patient 4 at initial presentation (**A**) and three days after ECTVS (**B**). The green circle indicates the circle along which the RNFL thickness is measured. The red and turquoise lines represent the estimated borders of the RNFL layer.

Disc edema was pronounced at presentation, with Frisen grades ranging from 3 to 5 and a mean RNFL thickness of 406 ± 93 µm on OCT. At the one-week follow-up, Frisen grading showed little change; however, OCT revealed a significant reduction in RNFL thickness, with a mean value of 309 ± 72 µm, corresponding to an average decrease of 97 µm compared with baseline (*p* < 0.001). By the last follow-up, the mean RNFL thickness had further decreased to 80 ± 31 µm, representing an average reduction of 326 µm from baseline (*p* < 0.00001). Figure 1 illustrates this reduction in patient 3, while Figure 2 demonstrates the corresponding findings in patient 4, highlighting the consistent post-procedural decline in RNFL thickness observed across the cohort.

Disc edema continued to improve gradually, and by the final visit, no patient had disc edema in either eye (Frisen grade 0). Optic atrophy was observed clinically in 4 of 10 eyes and on OCT (RNFL < 80 µm) in 6 of 10 eyes.

Acetazolamide dosage was tapered gradually following ECTVS, and all but two patients were off the medication by the final visit. Two patients experienced recurrent headaches during tapering, despite resolution of disc edema, and therefore continued acetazolamide at the last follow-up.

## 4. Discussion

In this study, we report a cohort of five patients with fulminant IIH and an imminent threat of blindness who were successfully treated with ECTVS. All patients underwent the procedure without complications or adverse effects, and visual function stabilized or improved in every treated eye.

Fulminant IIH is rare, accounting for approximately 2–3% of all IIH cases in most series [3]. Its clinical course is particularly alarming because of the rapid progression of visual loss, which almost invariably necessitates urgent surgical intervention [4]. Although CTVS has gained increasing acceptance as an effective treatment for IIH, there are relatively few published reports of its use specifically for fulminant cases. This may be partly explained by the fact that the largest fulminant IIH series to date, reported by Thambisetty et al., primarily involved patients treated with either ONSF or CSF shunting, and was published before CTVS became widely adopted [3].

CSF shunting procedures and ONSF are both regarded as effective in preserving, and in some cases restoring, vision in IIH. However, both are associated with considerable risks and long-term morbidities [10]. Although shunt surgeries have become safer over time, recent reviews report lifetime complication rates as high as 20%, mainly due to infection and shunt obstruction [5]. ONSF, while effective for halting vision loss, remains a technically demanding procedure with complication rates reported up to 25%, including orbital hemorrhage, infection, and even ischemic vision loss [6]. Moreover, ONSF does not address headache, a cardinal symptom of fulminant IIH, and up to 20% of patients later require a shunt procedure for headache control [10].

The SIGHT trial was designed to directly compare surgical modalities (VPS vs. ONSF vs. CTVS) but was terminated early due to low recruitment, making it unlikely that a comparable randomized trial in the much rarer fulminant IIH population will ever be completed. A recent expert debate emphasized that the choice of surgical modality should be guided by clinician experience and institutional expertise, as each option carries distinct advantages and drawbacks [11].

Patients with fulminant IIH require an immediate reduction of ICP to prevent irreversible optic nerve damage. Favorable results of CTVS in this setting have been previously described [12,13,14]. Our series represents the largest cohort focusing on neuro-ophthalmologic outcomes of fulminant IIH patients treated by a single interventional neurologist at a single tertiary center. Elder et al. were the first to publish a series of four fulminant IIH patients treated with ECTVS, but without long-term visual follow-up [13]. Zehri et al. reported a larger series of ten patients, focusing primarily on anatomical and neurosurgical aspects rather than standardized visual outcomes [14]. Our study applied a strict definition of fulminant IIH, requiring visual loss progression within four weeks of symptom onset.

In our cohort, all patients presented with marked disc edema, which gradually resolved in all eyes. Clinically, Frisen score reduction became evident only at the one-month follow-up. In contrast, OCT detected a significant decrease in RNFL thickness as early as one week post-procedure, averaging 98 µm. This suggests that OCT provides a more sensitive and objective assessment of treatment response compared with the qualitative Frisen grading scale, which requires substantial change to register improvement. Moreover, early OCT changes after ECTVS may have predictive value for subsequent recovery. In clinical practice, meaningful early RNFL reduction accompanied by stable visual function may indicate effective ICP reduction and allow tailored follow-up intensity, whereas concurrent GCL loss or functional decline should raise concern for axonal injury. Future prospective studies should standardize OCT timepoints and metrics (RNFL and GCL), correlate them with visual outcomes, and define predictive thresholds. Such work could establish OCT as a practical prognostic tool in fulminant IIH management.

However, while early RNFL thinning on OCT following ECTVS may indicate successful reduction of papilledema, it should be interpreted cautiously, as thinning can also represent irreversible axonal loss. Therefore, OCT changes must be correlated with long-term visual outcomes, particularly HVF recovery, to ensure accurate assessment of treatment success.

Complete resolution of disc edema was achieved in all eyes by the final visit. Optic atrophy was documented clinically in 4 of 10 eyes and by OCT (RNFL < 80 µm) in 6 of 10 eyes. All eyes with evidence of atrophy exhibited persistent visual field defects, a finding consistent with previous surgical series of fulminant IIH [14].

A descriptive comparison between eyes with and without optic atrophy revealed that patients who developed atrophy tended to have more severe baseline visual field loss and thicker RNFL values at presentation. These patients also had, on average, longer follow-up durations (≥18 months), suggesting that extended observation may increase the likelihood of detecting optic atrophy. In contrast, patients without atrophy generally presented with less advanced visual field damage and were followed for shorter periods (≤12 months). Although not a formal statistical analysis, these trends suggest that both baseline severity and follow-up duration may influence the incidence of optic atrophy.

While RNFL thinning was used in our study to indicate optic disc atrophy, it is acknowledged that ganglion cell layer thickness can be a more reliable marker for optic nerve degeneration. OCT angiography (OCT-A) could also have provided valuable information regarding optic nerve head perfusion, but was not available in this retrospective cohort. Furthermore, different OCT devices may generate automated reports that are not always reliable for detecting optic nerve swelling. For example, in our cohort, the device occasionally signaled normal values (“green” reports) despite evident edema. This underscores the importance of careful physician interpretation rather than reliance on automated analysis alone.

Interestingly, only one of our five patients was morbidly obese, and one had a normal BMI. These findings align with Elder et al., who reported only one morbidly obese patient among four fulminant cases [13]. This observation challenges the assumption that disease severity correlates directly with BMI and serves as a reminder that even mildly overweight or normal-weight patients remain at risk of fulminant vision-threatening IIH.

Different surgical techniques may be more appropriate depending on the clinical presentation and institutional expertise. ONSF is often favored when visual symptoms predominate and the disease course is less fulminant, as it directly relieves optic nerve head pressure but does not address headaches. CSF shunting remains a common option when both vision and headaches are problematic, but carries long-term risks of obstruction and infection. Emergent ECTVS, on the other hand, may be particularly suitable in fulminant cases with documented venous sinus stenosis, as it offers rapid and durable reduction of intracranial pressure while preserving vision. Our findings support the role of ECTVS in such urgent, vision-threatening scenarios.

The applicability of venous sinus stenting has also been demonstrated in recent studies and in special populations [15,16,17,18]. Regev et al. (2025) reported successful transverse sinus stenting for fulminant IIH during pregnancy, highlighting its feasibility even in this high-risk setting [16]. Pediatric series, though limited, suggest that the procedure can be performed safely in carefully selected patients [17], while isolated reports also describe its use in patients with significant comorbidities [18]. Taken together, these observations indicate that venous sinus stenting may represent a viable option across diverse clinical contexts, provided that multidisciplinary expertise and appropriate peri-procedural care are available.

Our study has several limitations. Its retrospective design resulted in some missing data, and no control group was available, which limits the strength of causal inference and precludes direct comparison with alternative treatment modalities. All patients presenting to our center with fulminant IIH during the study period elected to undergo ECTVS, which may introduce selection bias, particularly since SUMC is a tertiary referral center for endovascular neurology, where patients suitable for stenting are more likely to be referred. In addition, the small sample size limits generalizability, and we did not collect long-term functional outcomes such as quality of life or daily activity measures, which are important for fully assessing recovery beyond structural and visual parameters.

The small sample size represents a major limitation of this study and precludes formal statistical inference or generalization to broader populations. No power calculation was performed because this consecutive series reflects all eligible fulminant IIH cases treated with emergent venous sinus stenting at our center during the study period. As fulminant IIH is uncommon, assembling larger cohorts remains challenging. Therefore, our results should be interpreted as exploratory and hypothesis-generating, aimed at characterizing clinical trajectories and illustrating the potential role of OCT as an early, objective marker of treatment response.

Our stricter fulminant IIH definition, compared with prior reports such as Thambisetty et al. [3], was chosen to reflect real-world decision-making for emergent venous sinus stenting and to improve internal validity. However, this choice may reduce direct comparability with series using broader criteria and may bias our cohort toward greater baseline severity (e.g., worse MD, higher likelihood of subsequent atrophy). These differences should be considered when interpreting external generalizability across studies.

It should be noted that our institutional cutoff for stent placement was a trans-stenotic pressure gradient of ≥8 mmHg. While other groups have reported using thresholds ranging from 6 to 10 mmHg, our criterion represents a balance between procedural efficacy and safety. This variability highlights the need for standardization and should be considered when generalizing our results to other centers.

Despite these limitations, our findings suggest that ECTVS is a safe and effective treatment for fulminant IIH, providing rapid visual recovery and papilledema resolution. This approach may represent a valuable alternative to traditional surgical interventions and has the potential to reduce the risk of permanent vision loss in this high-risk population. Larger, multicenter studies with longer follow-up are warranted to further validate ECTVS as a standard treatment option for fulminant IIH.

## 5. Conclusions

ECTVS appears to be a safe and effective vision-preserving treatment for fulminant idiopathic intracranial hypertension, offering rapid reduction of intracranial pressure and early neuro-ophthalmologic improvement. OCT enables sensitive, objective monitoring and may serve as an early predictor of treatment success, supporting its integration into post-procedural follow-up protocols. Larger, prospective studies are warranted to confirm these findings and to further define the prognostic role of OCT in this setting.

## Figures and Tables

**Table 1:** Baseline Clinical Characteristics of Patients at Presentation.

Patient	Age (Years)	BMI	Visual and Other Symptoms	LP OP (mmH_2_O)	Initial Treatment	Trans Stenotic Gradient (R/L) (mmH_2_O)
1	17	26	Blurred vision, ear pain, pulsatile tinnitus	380	Acetazolamide 3000 mg/day +IVMP	14/13
2	18	22	Blurred vision, TVO	500	Acetazolamide 2000 mg/day +IVMP	32/12
3	18	38	Blurred vision, TVO, neck pain, nausea	500	Acetazolamide 3000 g/day +IVMP	19/10
4	21	30	Pulsatile tinnitus	510	Furosemide 80 mg	30/32
5	20	48	Blurred vision, TVO, neck pain	380	Acetazolamide 2000 mg/day +IVMP	30/27

BMI—body mass index; LP OP—lumbar puncture opening pressure; R/L—right/left; TVO—transient visual obscurations; IVMP—intravenous methylprednisolone.

**Table 2:** Neuro-Ophthalmologic Assessments Before and After ECTVS.

		Patients				
		1	2	3	4	5
BCVA (R/L)	Presentation	0/0	0/0	1.3/1	0.3/0.2	1/0
	1W post-op	0/0	0/0	1.3/0.7	0/0.1	1/0
	1M post-op	0/0	0/0	0.3/0.1	0/0.1	0.4/0
	Final visit	0/0	0/0	0.2/0	0/0	0.4/0
Color plates * (R/L)	Presentation	12/12	12/12	10/12	12/12	9/12
	1W post-op	12/12	12/12	12/12	12/12	10/12
	1M post-op	12/12	12/12	12/12	12/12	10/12
	Final visit	12/12	12/12	12/12	12/12	11/12
HVF MD (R/L)	Presentation	N/A	N/A	−31/−32	−21/−6	−32/−16
	1W post-op	−22/−26	N/A	−30/−32	−13/−2	−32/−17
	1M post-op	−5/−15	−15/−18	−30/−32	−13/−2	−32/−17
	Final visit	−3/−8	−4/−4	−29/−23	−12/−3	−25/−9
Disc edema Frisen grade (R/L)	Presentation	4/4	5/5	5/5	4/3	5/3
	1W post-op	4/4	5/4	5/5	4/3	5/3
	1M post-op	3/3	2/2	3/3	2/2	2/1
	Final visit	0/0	0/0	atrophy/atrophy	atrophy/0	atrophy/0
OCT RNFL thickness, microns (R/L)	Presentation	442/441	237/248	490/402	480/415	497/405
	1W post-op	411/403	210/217	268/273	357/344	338/264
	1M post-op	190/183	141/156	93/101	120/168	177/194
	Final visit	98/72	120/124	45/38	54/106	62/79
Follow-Up after ECTVS (months)		9	8	26	15	6

BCVA—Best Corrected Visual Acuity measured in logMAR; R/L—right/left; MD—mean deviation; HVF—Humphrey Visual Field; OCT—optical coherence tomography, RNFL—retinal nerve fiber layer; ECTVS—emergent cerebral transverse venous stenting, N/A—not available. * Ishihara color plates with 12 numbers.

## Data Availability

The original contributions presented in this study are included in the article. Further inquiries can be directed to the corresponding author.
